# Supplementary material for: A Novel Chemotherapy Combination to Enhance Proteotoxic Cell Death in Hepatocellular Carcinoma Experimental Models Without Killing Non-Cancer Cells
Source: Int J Mol Sci. 2025 Jul 12;26(14):6699. doi: 10.3390/ijms26146699 (PMC12294793; doi:10.3390/ijms26146699)
Supplement: Supplementary file 1 [file ijms-26-06699-s001.zip › Supplementary Figures.pdf]

Supplementary Tables S1, S2
Supplementary Figures S1-S12

A Novel Chemotherapy Combination to Enhance Proteotoxic Cell Death in Hepatocellular Carcinoma Without Killing Non-Cancer Cells

Carlos Perez-Stable, Alicia de las Pozas, Teresita Reiner, Medhi Wangpaichitr, Jose Gomez, Manojavan Nagarajan, Robert Foster, Daren Ure

Figure S1

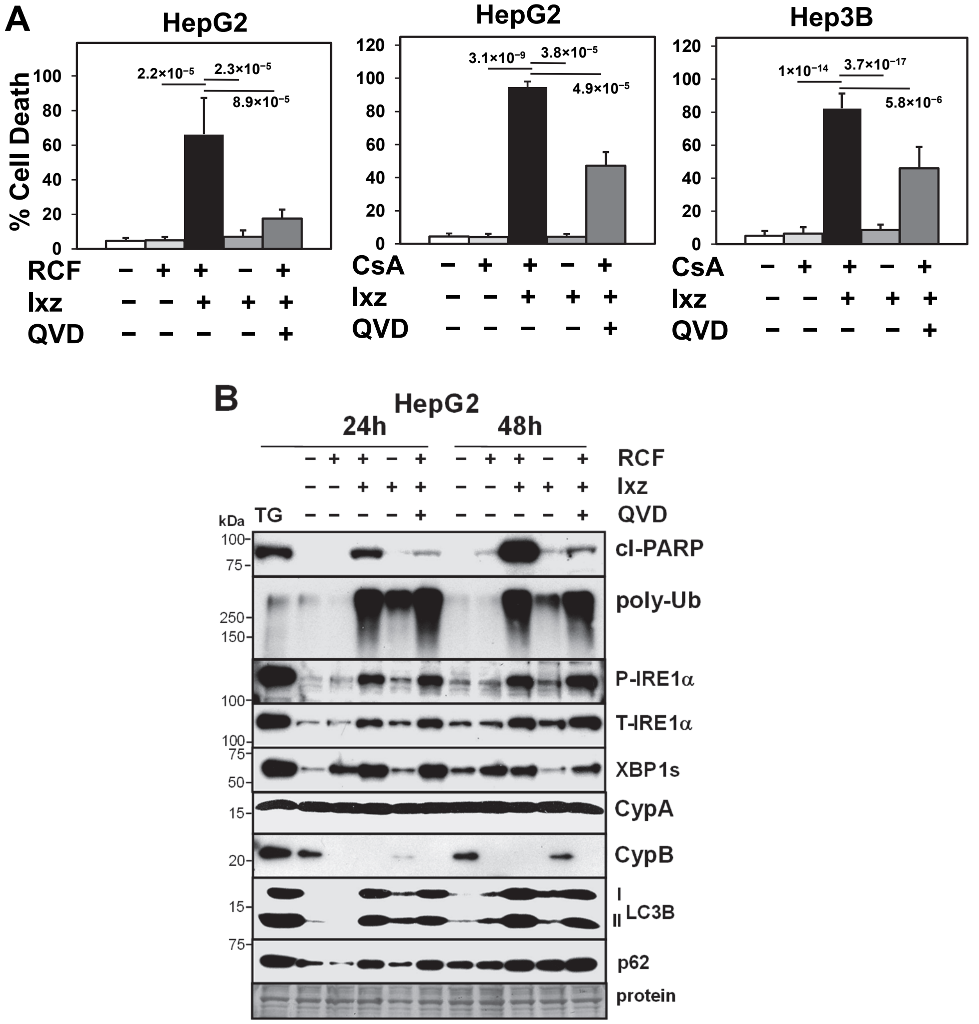

Figure S1. RCF or CsA + Ixz combination enhances apoptotic cell death in HepG2 and Hep3B HCC cells. **(A)** Trypan blue exclusion assay showed significantly higher cell death in RCF (2 μM) or CsA (10 μM) + Ixz (9708; 100 nM with RCF and 50 nM with CsA) treated HepG2 and Hep3B (72h) compared to RCF/CsA, Ixz, and control treated cells. Addition of apoptosis inhibitor QVD (10 μM) significantly decreased RCF/CsA + Ixz cell death in HepG2 and Hep3B. *P* values are shown near the bars. **(B)** Western blot analysis showed increased cl-PARP, poly-Ub, IRE1α (P/total [T]), XBP1s, LC3B, and p62 (suggesting inhibition of autophagy) in RCF (2 μM) + Ixz (9708; 100 nM) treated HepG2 (24, 48h) compared to RCF, Ixz, and control treated cells. CypB decreased with RCF and RCF + Ixz (24, 48h) and no changes were noted with CypA. Addition of QVD to RCF + Ixz only decreased cl-PARP. TG (thapsigargin, 10 nM) is positive control for ER stress. Size of molecular weight markers in kDa shown to the left. Protein refers to Coomassie blue stain after all immunological analysis was completed.

Table S1

Table S1 RCF + Ixz synergistically inhibits Hep3B and PLC HCC cells

	RCF (μM)	FA	Ixz (nM)	FA	FA (RCF+Ixz)	CI
Hep3B	5	0.01	10	0.01	0.71	0.45
	5	0.01	15	0.44	0.82	0.46
	5	0.01	25	0.68	0.93	0.44
PLC	5	0.00	10	0.00	0.61	0.35
	5	0.00	15	0.23	0.79	0.33
	5	0.00	25	0.66	0.97	0.25

Cell proliferation assay showed that various combinations of RCF + Ixz (2238) synergistically inhibited Hep3B and PLC as determined by combination index (CI). FA, fraction affected refers to inhibition (no inhibition control=0; 100% inhibition=1.0).

Table S2

Table S2 RCF or CsA + Ixz synergistically inhibits HepG2 and Hep3B HCC cells

	RCF (μM)	FA	Ixz (nM)	FA	FA (RCF+Ixz)	CI
HepG2	2	0.00	75	0.06	0.32	0.52
	2	0.00	100	0.07	0.61	0.39
	2	0.00	125	0.08	0.95	0.18
	2	0.00	150	0.22	0.95	0.19

	CsA (μM)	FA	Ixz (nM)	FA	FA (CsA+Ixz)	CI
HepG2	10	0.06	25	0.00	0.52	0.17
	10	0.06	50	0.00	0.70	0.25
	10	0.06	75	0.06	0.96	0.22
Hep3B	10	0.00	25	0.00	0.54	0.23
	10	0.00	50	0.00	0.73	0.40
	10	0.00	75	0.41	0.96	0.47

Cell proliferation assay showed that various combinations of RCF or CsA + Ixz (9708) synergistically inhibited HepG2 and Hep3B as determined by combination index (CI). FA, fraction affected refers to inhibition (no inhibition control=0; 100% inhibition=1.0).

Figure S2

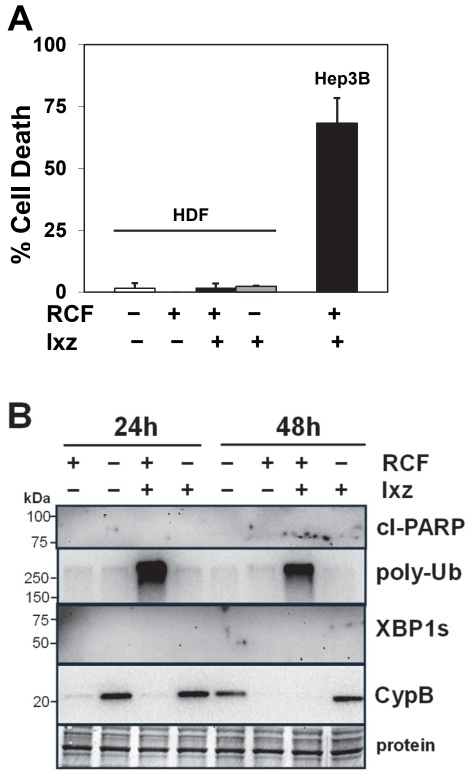

Figure S2. RCF + Ixz has no effect on cell death in non-cancer HDF cells. (A) Trypan blue exclusion assay showed RCF (5 μM) + Ixz (15 nM) did not increase cell death in HDF cells compared to RCF, Ixz, and control treated cells. In contrast, RCF + Ixz greatly increased cell death in Hep3B (68%). **(B)** Western blot analysis showed RCF + Ixz did not increase cl-PARP or XBP1s but increased poly-Ub and decreased CypB in HDF. Size of molecular weight markers in kDa shown to the left. Protein refers to Coomassie blue stain after all immunological analysis was completed.

Figure S3

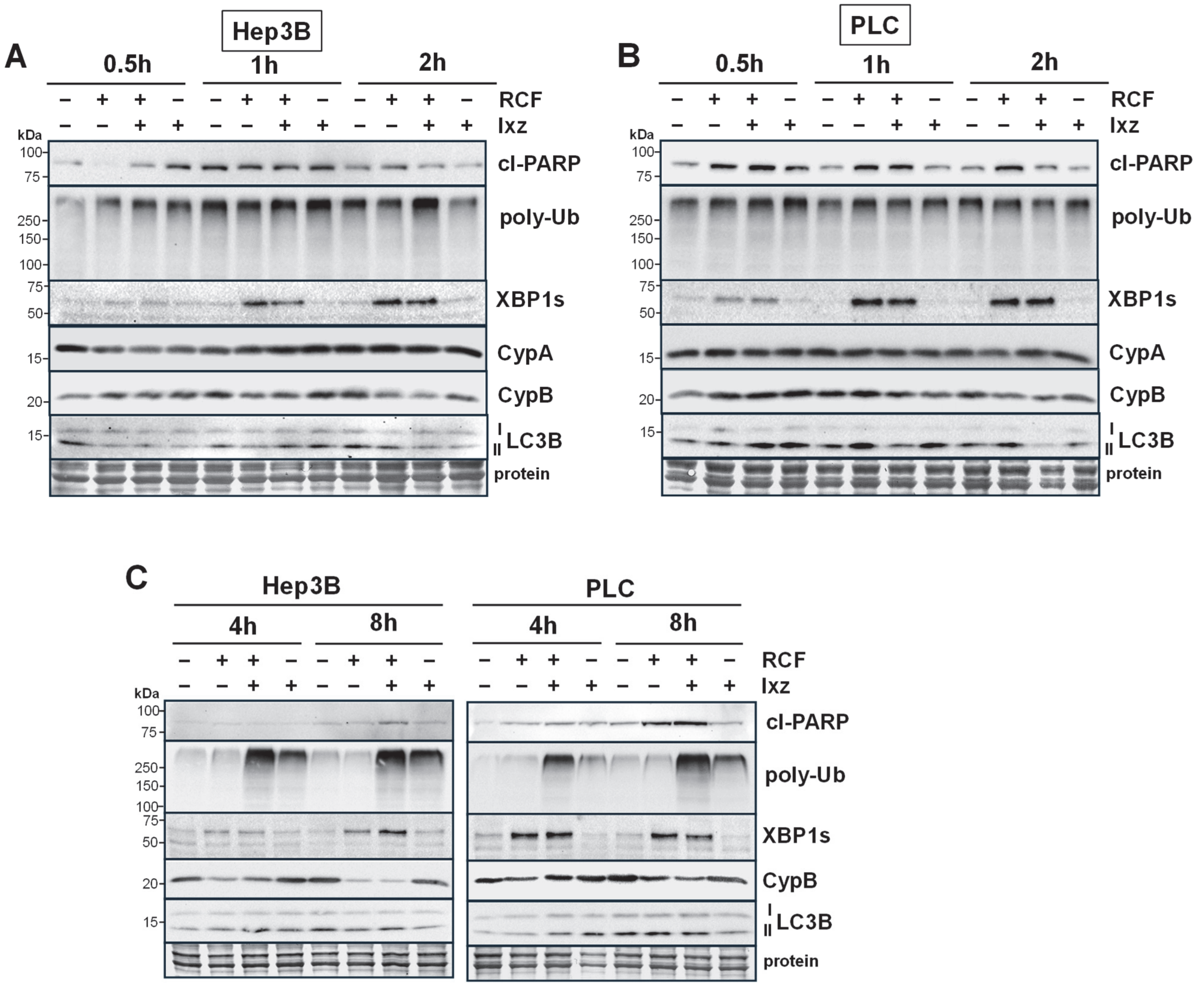

Figure S3. Early increase in XBP1s in RCF alone and RCF + Ixz treated HCC cells. Western blot analysis showed early increased XBP1s in RCF (5 μM) alone and RCF + Ixz (2238; 15 nM) treated Hep3B **(A)** and PLC **(B)** (1, 2h) compared to RCF, Ixz, and control treated cells. No clear differences were noted for cl-PARP, poly-Ub, CypA/B, and LC3B. **(C)** At later times, RCF + Ixz increased poly-Ub (4h; also increased by Ixz alone) and cl-PARP (8h) and decreased CypB (4, 8h) in Hep3B and PLC. No clear differences were noted with LC3B. Size of molecular weight markers in kDa shown to the left. Protein refers to Coomassie blue stain after all immunological analysis was completed.

Figure S4

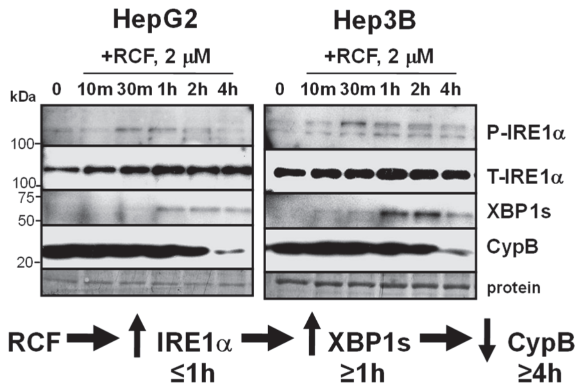

Figure S4. Immediate early effects of RCF alone on HCC cells. Western blot analysis showed an immediate early increase in IRE1α (P/T[total]) (10, 30m) followed by XBP1s (1h) and decreased CypB (4h) in RCF (2 μM) alone treated HepG2 and Hep3B. Size of molecular weight markers in kDa shown to the left. Protein refers to Coomassie blue stain after all immunological analysis was completed. Timeline of RCF molecular changes are shown below Western blot.

Figure S5

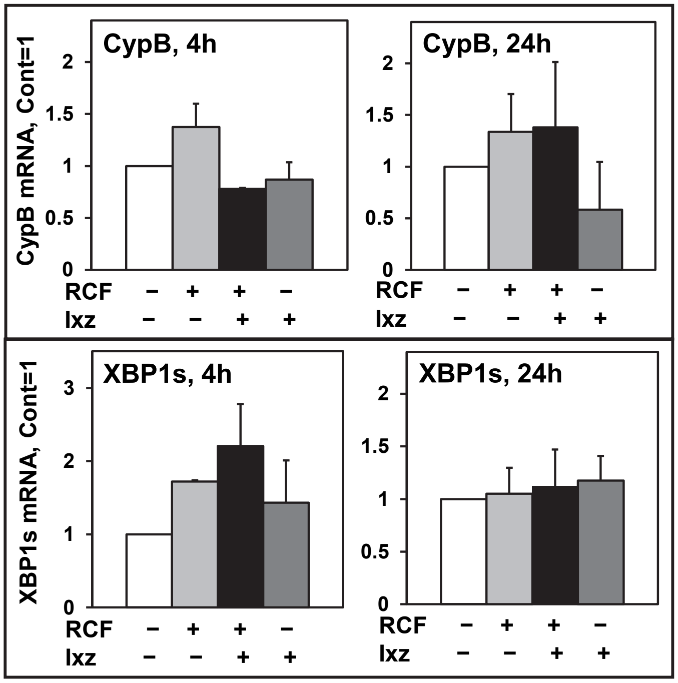

Figure S5. CypB and XBP1s qPCR analysis of RCF + Ixz treated Hep3B HCC cells. Top panel: qPCR analysis showed no clear differences in CypB mRNA (despite decreased protein) in RCF (5 µM) + Ixz (9708; 25 nM) compared to RCF, Ixz, and control treated Hep3B (4, 24h; *n*=4, 2 independent experiments). Bottom panel: XBP1s mRNA was higher in RCF and RCF + Ixz at 4h but not 24h. Values were normalized to control =1.

Figure S6

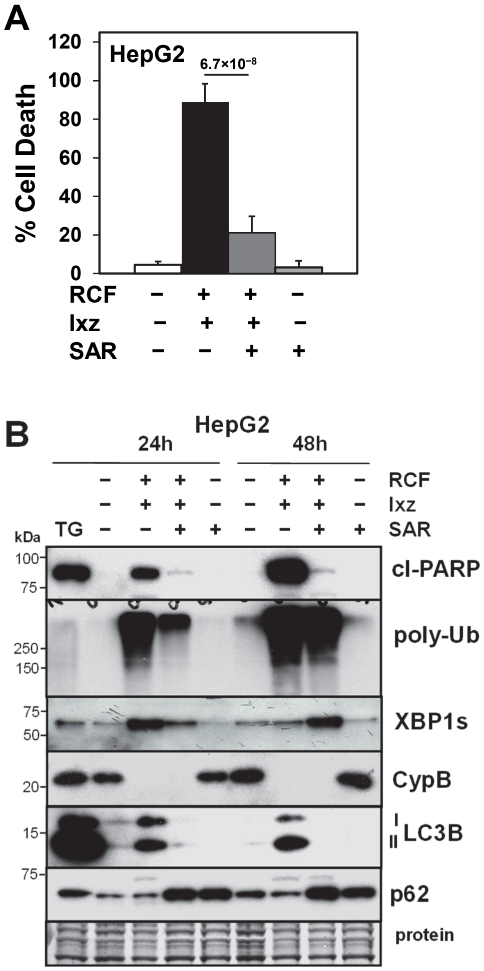

Figure S6. Autophagy inhibitor SAR405 blocks RCF + Ixz increase in apoptotic cell death in HepG2. (A) Trypan blue exclusion assay showed addition of SAR (1 μM) to RCF (2 μM) + Ixz (9708; 100 nM) significantly decreased cell death in HepG2 cells (72h). *P* value shown above the bar. **(B)** Western blot analysis showed SAR decreased cl-PARP, poly-Ub, XBP1s (24h), LC3B, and increased p62 (suggesting inhibition of autophagy) in RCF + Ixz treated HepG2 (24, 48h). There were no clear differences in CypB. Size of molecular weight markers in kDa shown to the left. Protein refers to Coomassie blue stain after all immunological analysis was completed.

Figure S7

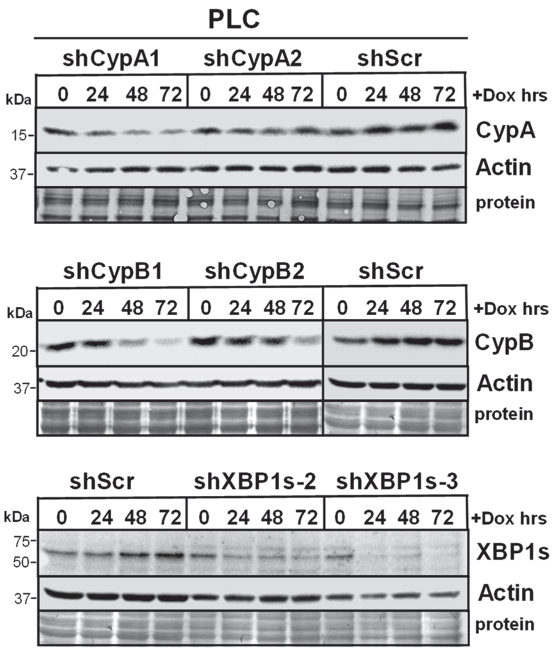

Figure S7. Inducible knockdowns of CypA, CypB, and XBP1s in PLC HCC cells. Western blot analysis of PLC/shCypA-1, -2, shScr (top blot), PLC/shCypB-1, -2, shScr (middle blot), and PLC/shXBP1s-2, -3, shScr (bottom blot) treated with Dox (100 ng/ml) for 24, 48, and 72h. Results showed decreased CypA (top), CypB (middle), and XBP1s (bottom) compared to control (0h) and actin. Dox treatment of PLC/shScr negative control showed no clear differences in CypA, B, XBP1s, or actin. The vertical line in the middle blot indicates separate blots. Size of molecular weight markers in kDa shown to the left. Protein refers to Coomassie blue stain after all immunological analysis was completed.

Figure S8

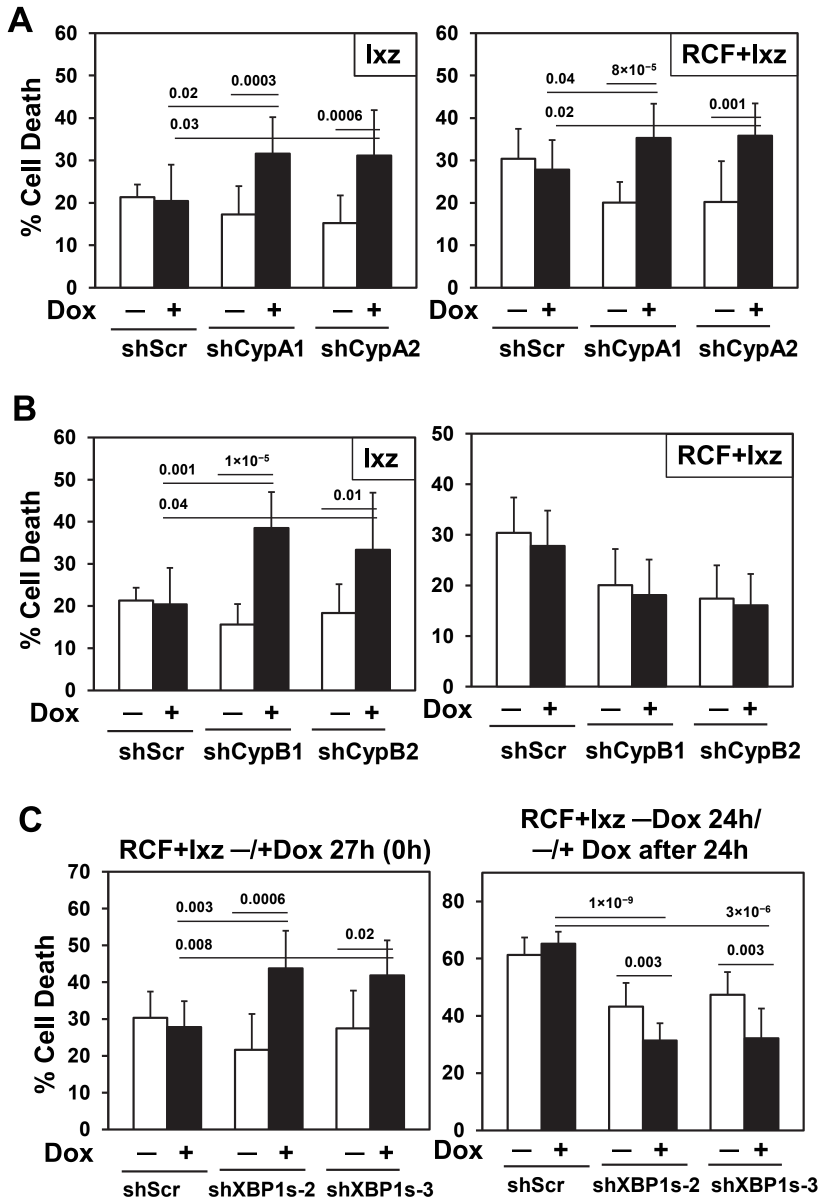

Figure S8. Inducible knockdowns: CypA and CypB are pro-survival whereas XBP1s is pro-survival early but pro-cell death later in RCF + Ixz treated PLC HCC cells. Trypan blue exclusion assays showed addition of Dox (+) to induce knockdown of CypA **(A)** increased Ixz (2238; 15 nM) (72h) and RCF (5 µM) + Ixz (15 nM) (27h) cell death in PLC/shCypA-1 and -2. Addition of Dox to induce knockdown of CypB **(B)** increased cell death in Ixz but not in RCF + Ixz treated PLC/shCypB-1 and -2. Addition of Dox to induce knockdown of XBP1s **(C)** at time 0h increased cell death in RCF + Ixz treated PLC/shXBP1s-2 and -3 (27h). However, addition of Dox 24h after RCF + Ixz decreased cell death after another 24h. In all cases, addition of Dox to PLC/shScr negative control cells did not result in differences in cell death. In addition, no Dox (−) cell death was similar to Scr negative control cells. In the shorter RCF + Ixz (27h) treatments, cells were pretreated with Dox for 48h. *P* values were shown above the bars.

Figure S9

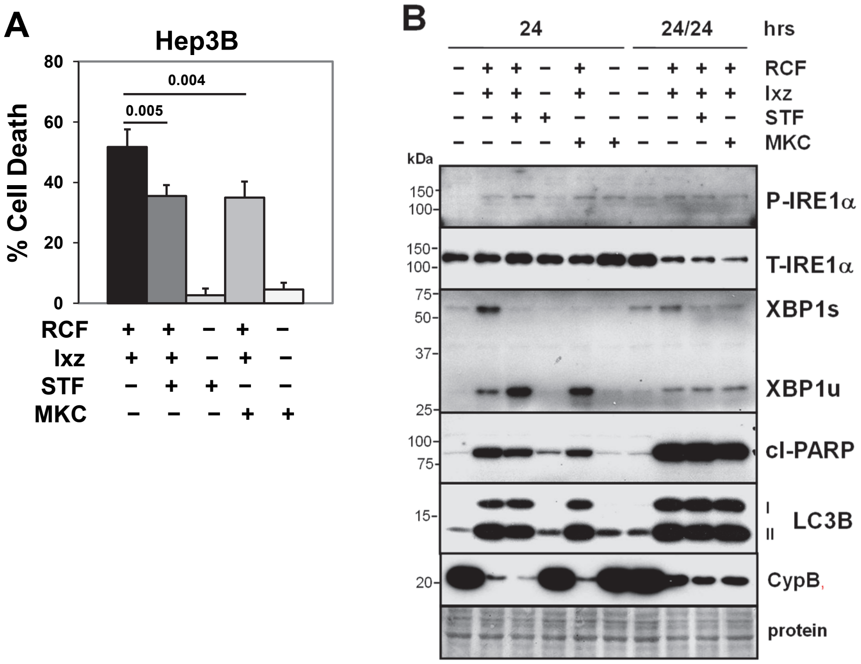

Figure S9. IRE1α inhibitors block RCF + Ixz cell death in Hep3B by decreasing XBP1s. (A) Trypan blue exclusion assays showed addition of IRE1α inhibitors STF-083010 (50 μM) or MKC-3946 (20 μM) after 24h of RCF (2 μM) + Ixz (100 nM) decreased cell death. *P* values were shown above the bars. **(B)** Western blot analysis showed addition of IRE1α inhibitors STF (50 μM) or MKC (20 μM) at the start of RCF (2 μM) + Ixz (100 nM) for 24h decreased XBP1s and increased XBP1u (unspliced precursor to XBP1s). Similar results were obtained when STF or MKC were added 24h after the start of RCF + Ixz (24/24). There were no clear differences in IRE1α (P/T), cl-PARP, LC3B, or CypB. Size of molecular weight markers in kDa shown to the left. Protein refers to Coomassie blue stain after all immunological analysis was completed.

Figure S10

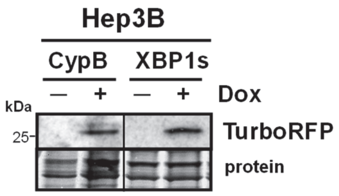

Figure S10. Turbo RFP (lentivirus plasmid protein) is induced by Dox. Western blot analysis of Hep3B/CypB and Hep3B/XBP1s treated with RCF (5 μM) + Ixz (15 nM) for 72h (CypB) or 48h (XBP1s) −/+Dox. Results showed the addition of Dox induced TurboRFP protein. Vertical line refers to removal of lanes between CypB and XBP1s samples from the same blot. The size of molecular weight marker in kDa is shown to the left. Protein refers to Coomassie blue stain after immunological analysis was completed.

Figure S11

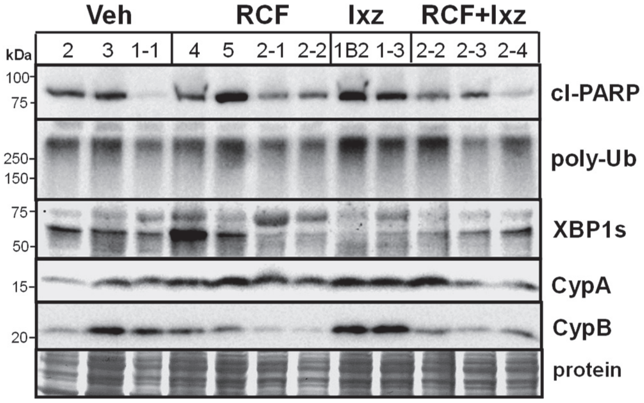

Figure S11. Protein differences in RCF + Ixz treated Hep3B xenograft tumors. Western blot analysis of Hep3B xenograft tumors from representative mice treated with RCF + Ixz (2-2, 2-3, 2-4) compared to RCF (4, 5, 2-1, 2-2), Ixz (1B2, 1-3), and vehicle (Veh) controls (2, 3, 1-1). Results showed the only possible difference detected between the different groups was decreased CypB in RCF and RCF + Ixz. No clear differences were noted with cl-PARP, poly-Ub, XBP1s, or CypA. Size of molecular weight markers in kDa shown to the left. Protein refers to Coomassie blue stain after immunological analysis was completed.

Figure S12

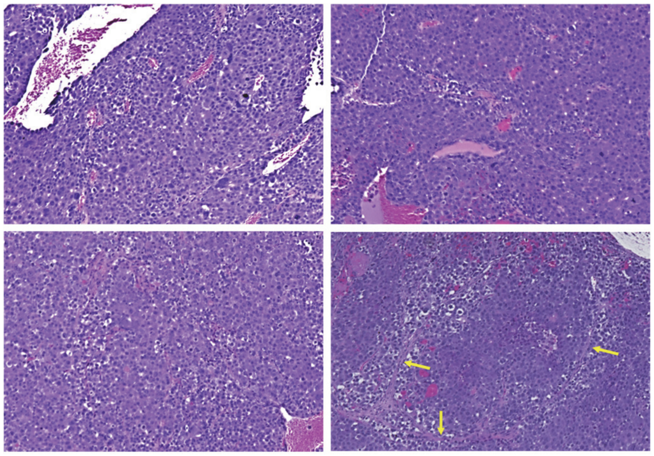

Figure S12. Possible increase in vacuolated cells in RCF + Ixz Hep3B xenograft tumors. H&E staining (x40) of representative Hep3B xenograft tumors showed very little differences with the possible exception in RCF + Ixz of increased vacuolated cells (proteotoxic stress) near blood vessels (yellow arrows) compared to vehicle, RCF, and Ixz.
