# Supplementary material for: A Novel Chemotherapy Combination to Enhance Proteotoxic Cell Death in Hepatocellular Carcinoma Experimental Models Without Killing Non-Cancer Cells
Source: Int J Mol Sci. 2025 Jul 12;26(14):6699. doi: 10.3390/ijms26146699 (PMC12294793; doi:10.3390/ijms26146699)
Supplement: Supplementary file 1 [file ijms-26-06699-s001.zip › Uncropped Western Blots.pdf]

A Novel Chemotherapy Combination to Enhance Proteotoxic Cell Death in Hepatocellular Carcinoma Experimental Models Without Killing Non-Cancer Cells

Carlos Perez-Stable, Alicia de las Pozas, Teresita Reiner, Medhi Wangpaichitr, Jose Gomez, Manojavan Nagarajan, Robert T. Foster, Daren R. Ure

Uncropped Western Blots: Figures 1-5

1. **Blots were cut horizontally to analyze high, medium, or low molecular weight target proteins.**
2. **Regions used in figures are contained within red rectangles.**

Figure 1B

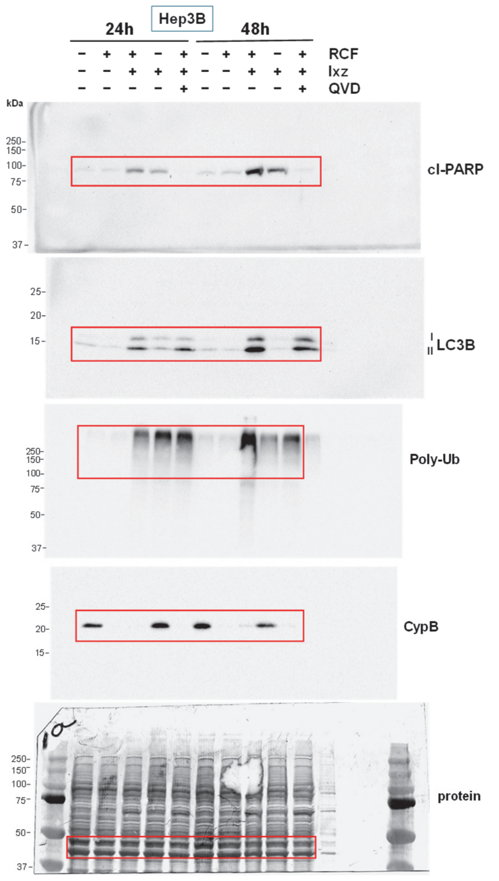

Western blot #1: 8/11/2023

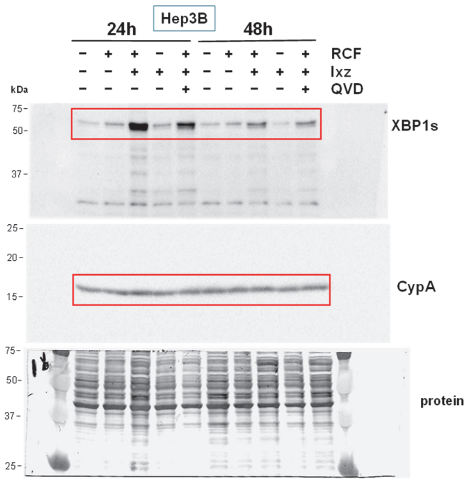

Western blot #1: 8/31/2023

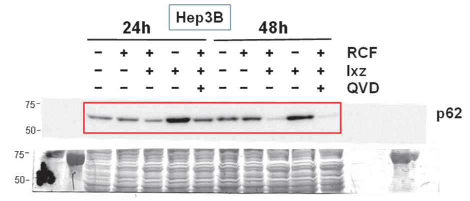

Western blot #1: 10/12/2023

Figure 1C

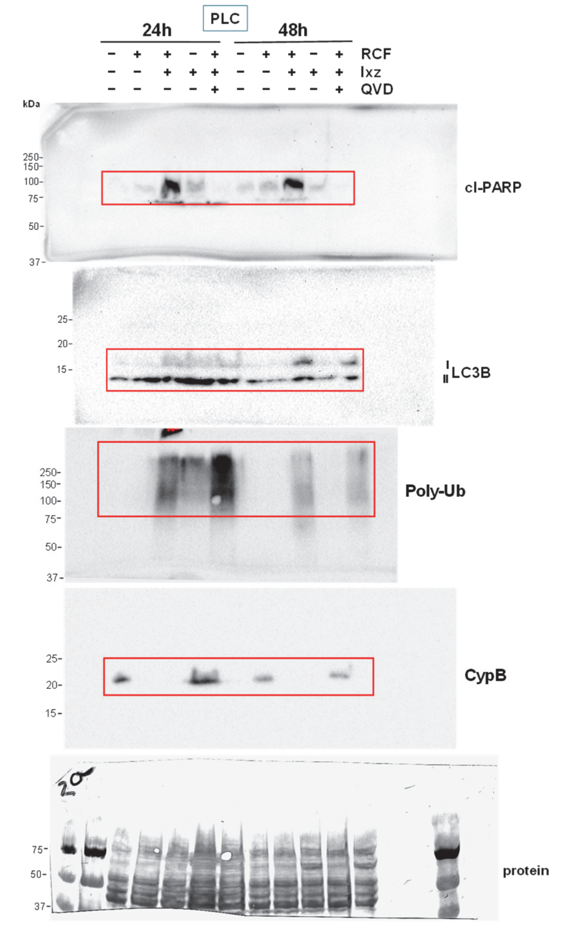

Western blot #2: 8/11/2023

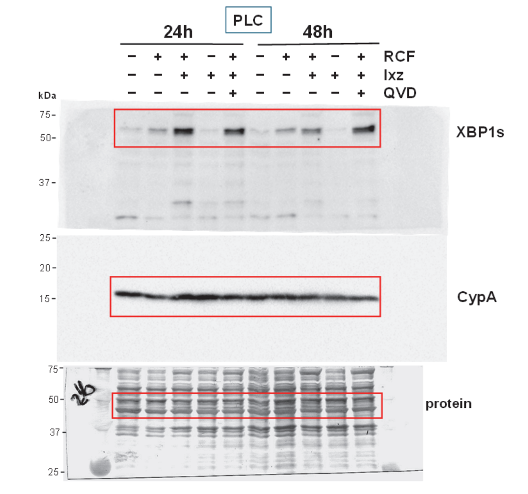

Western blot #2: 8/31/2023

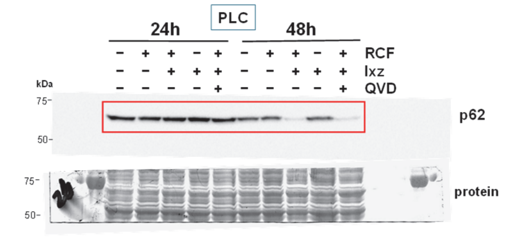

Western blot #2: 10/12/2023

Figure 1E

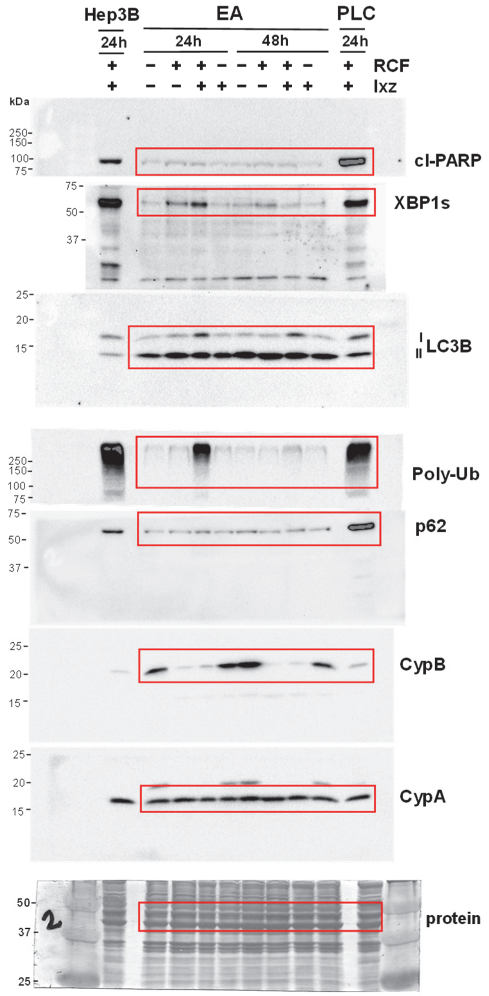

Western blot 5/10/2024

Figure 2B

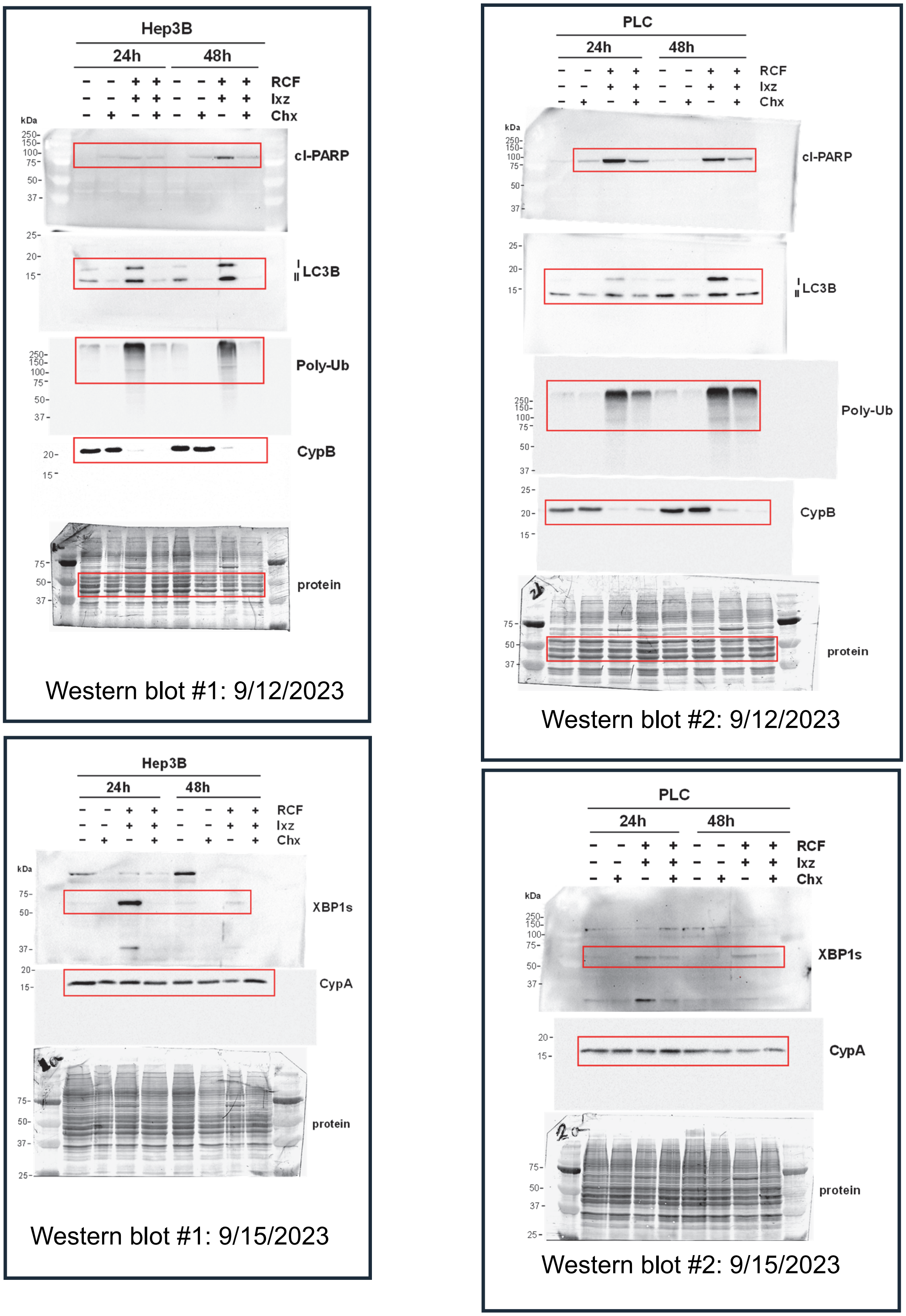
Hep3B
24h
48h
RCF
Ixz
Chx
kDa
cl-PARP
LC3B
Poly-Ub
CypB
protein
Western blot #1: 9/12/2023
PLC
cl-PARP
LC3B
Poly-Ub
CypB
protein
Western blot #2: 9/12/2023
Hep3B
XBP1s
CypA
protein
Western blot #1: 9/15/2023
PLC
XBP1s
CypA
protein
Western blot #2: 9/15/2023

Figure 2C

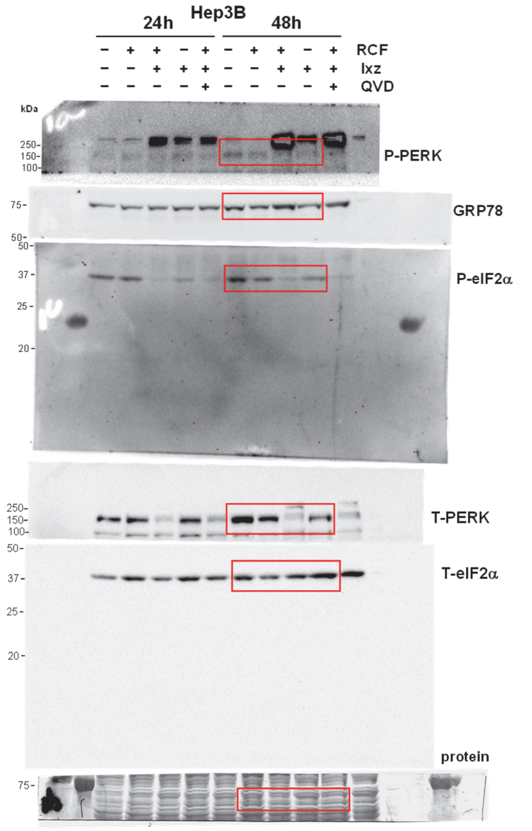

Western blot #1: 10/12/2023

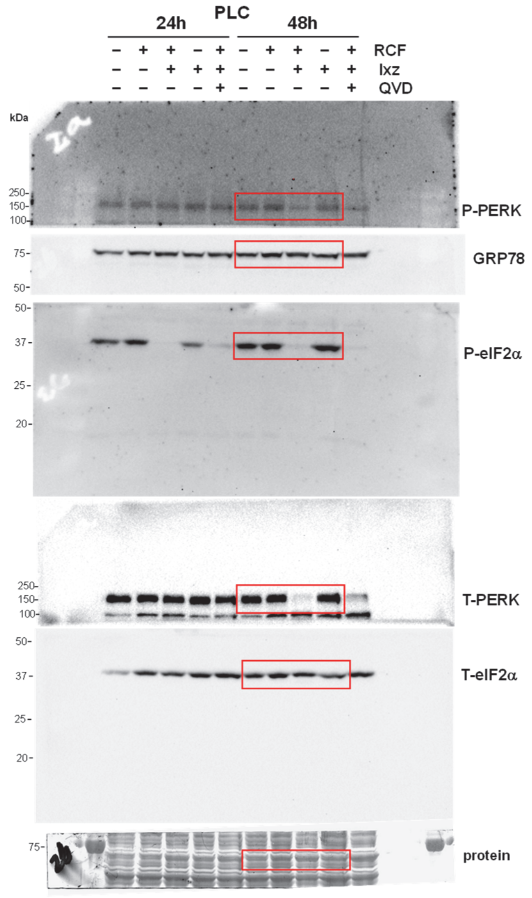

Western blot #2: 10/12/2023

Figure 2D

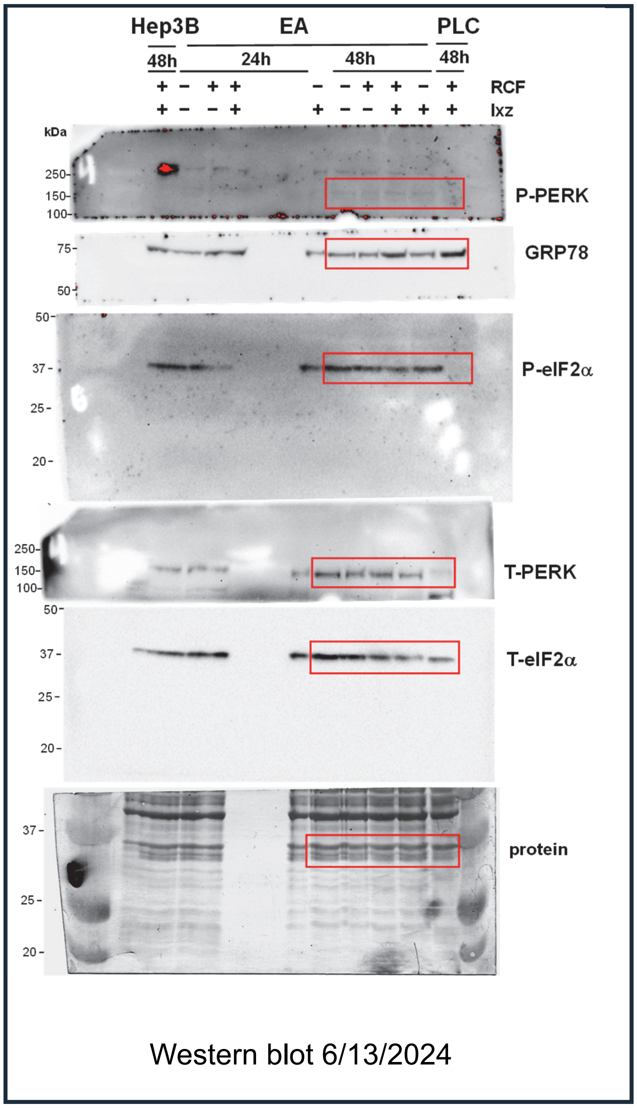
Hep3B
EA
PLC
48h
24h
48h
48h
RCF
Ixz
kDa
250-
150-
100-
P-PERK
75-
50-
GRP78
50-
37-
25-
20-
P-eIF2α
250-
150-
100-
T-PERK
50-
37-
25-
20-
T-eIF2α
37-
25-
20-
protein
Western blot 6/13/2024

Figure 2F

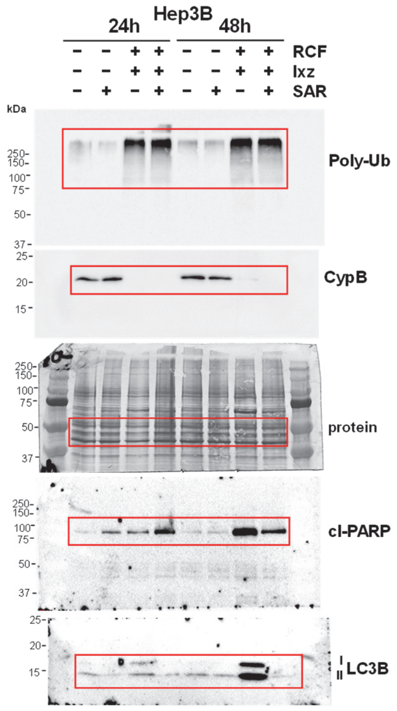

Western blot #1: 10/17/2023

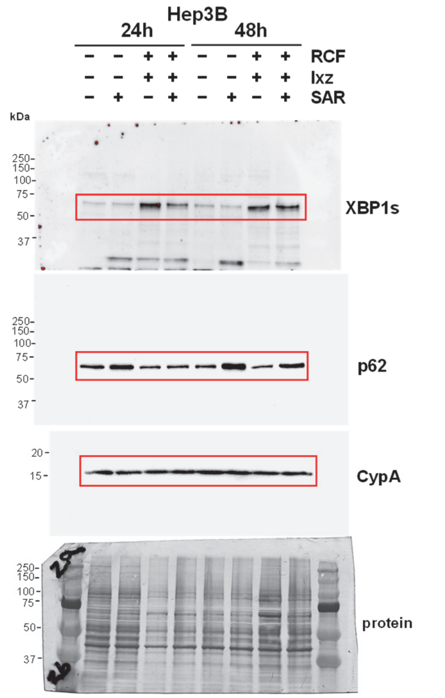

Western blot #2: 10/17/2023

Figure 3A

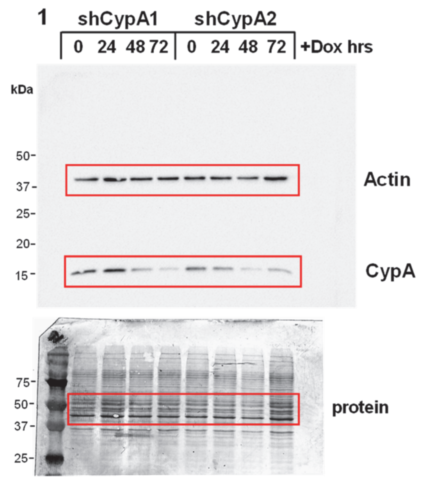

Western blot 11/16/2022

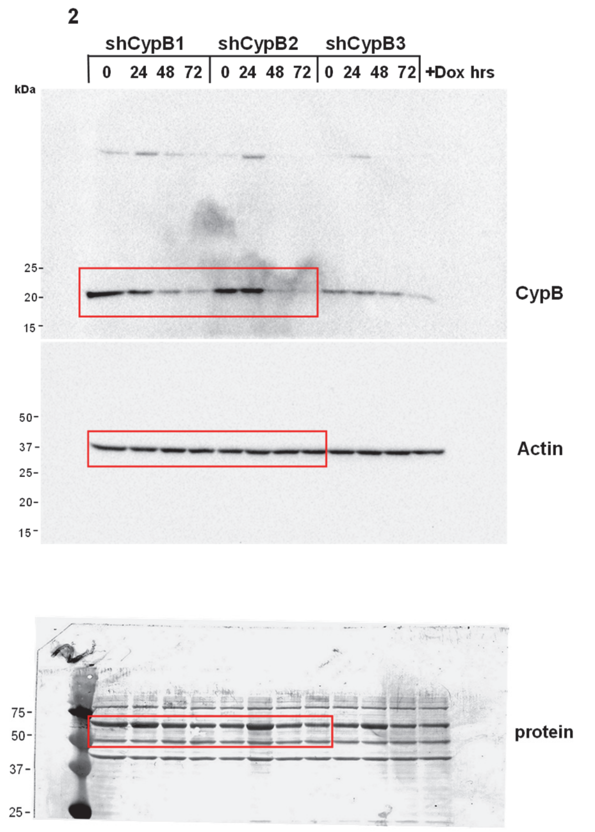

Western blot 11/29/2022

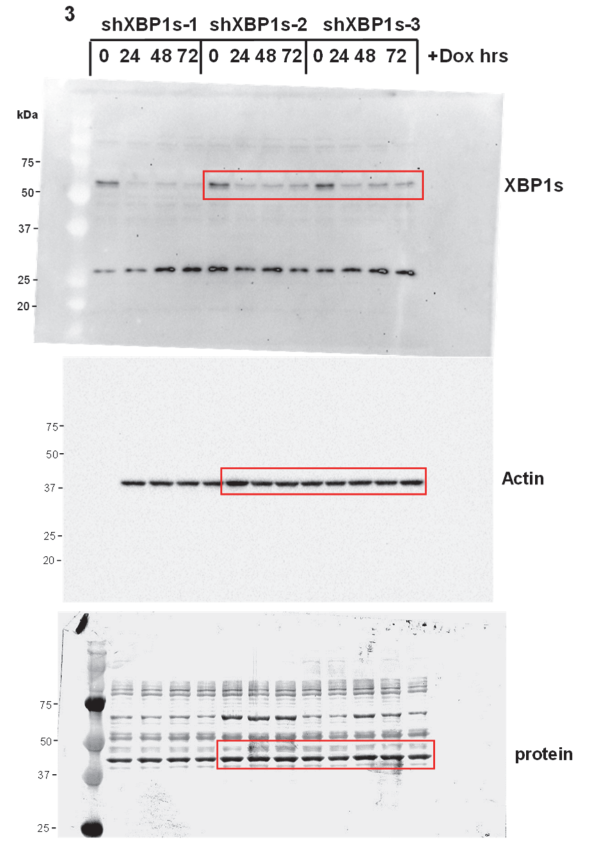

Western blot 12/1/2022

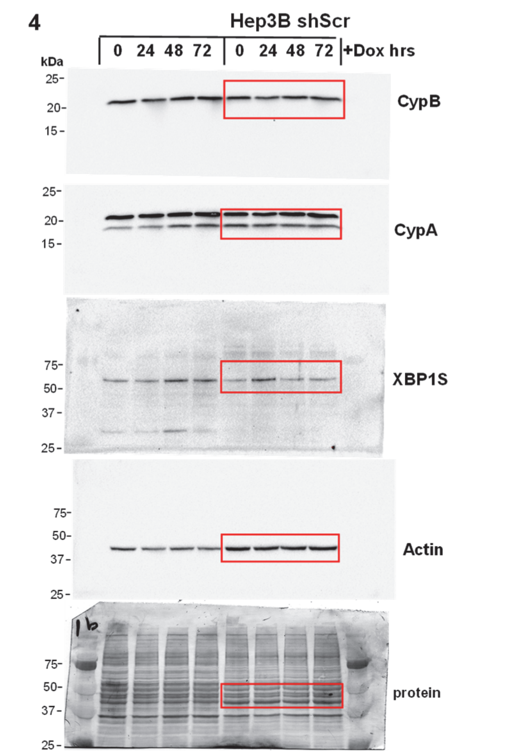

Western blot 7/31/2024

Figure 4A

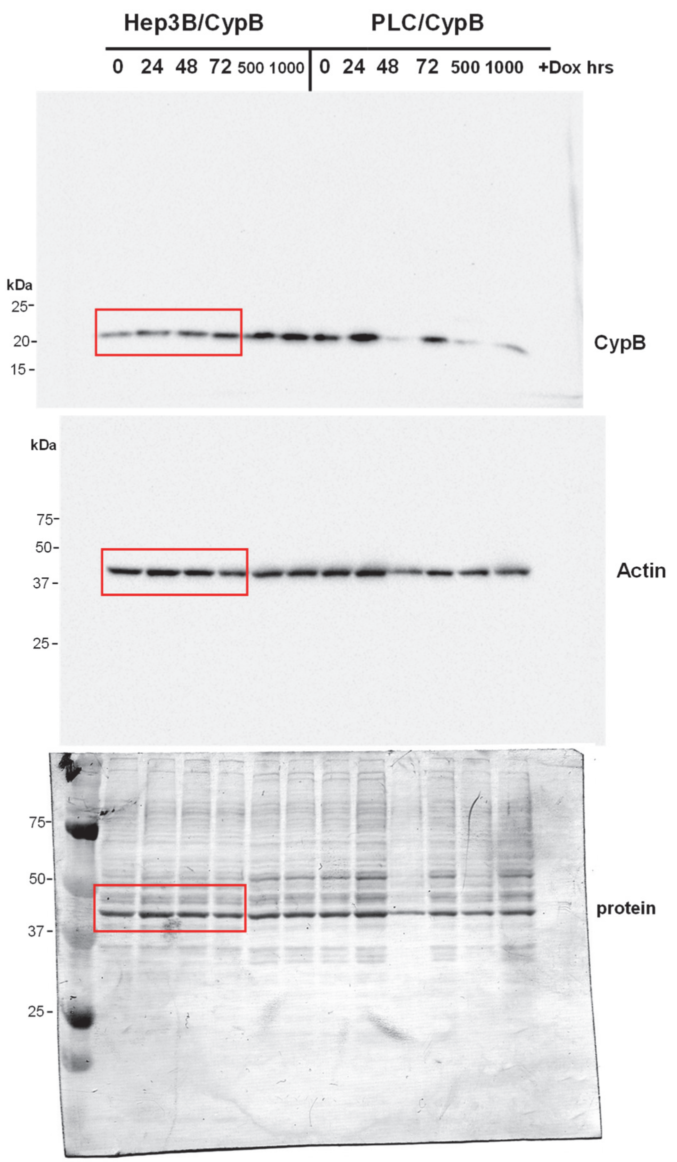

Western blot 8/8/2023

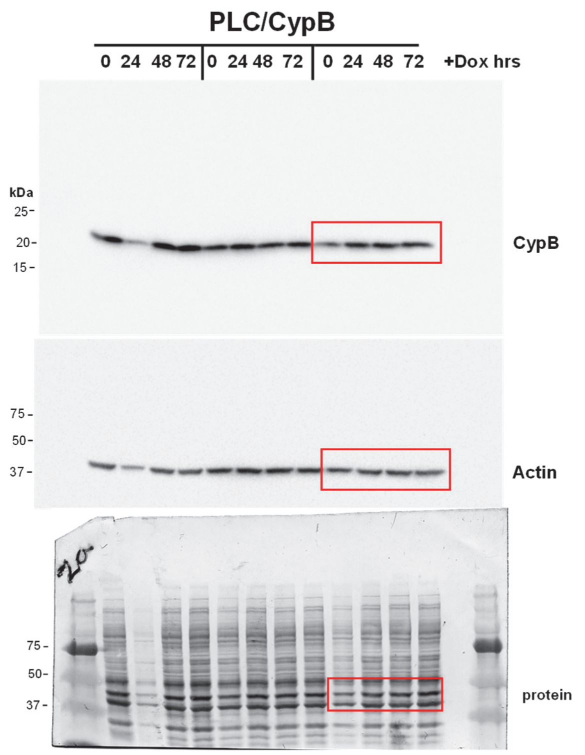

Western blot 9/22/2023

Figure 4C

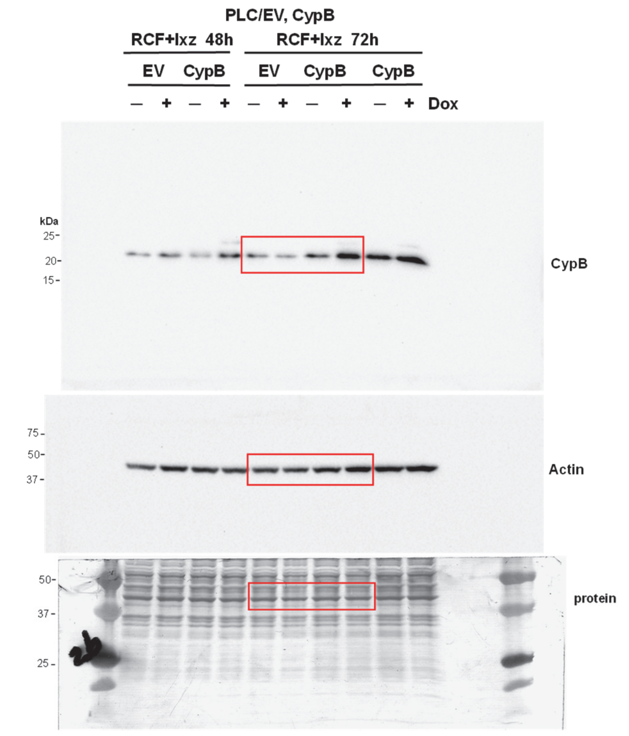

Western blot #2: 9/29/2023

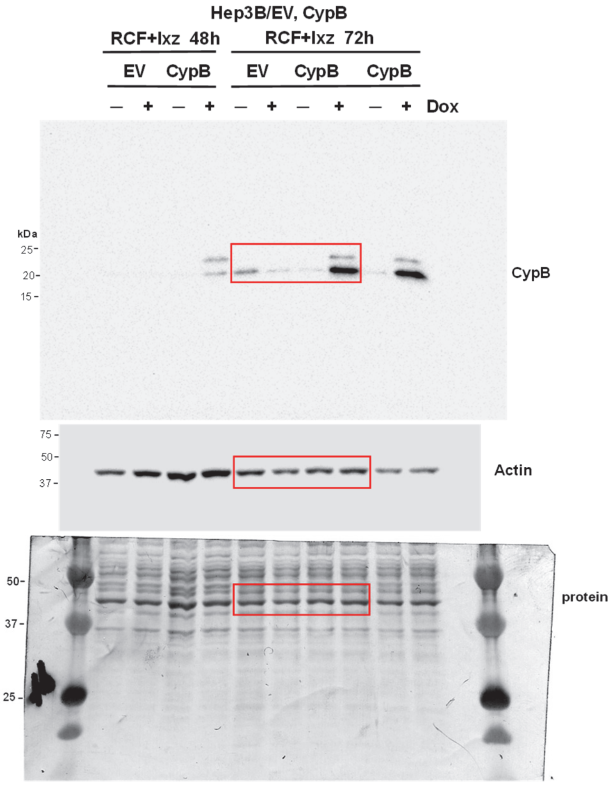

Western blot #1: 9/29/2023

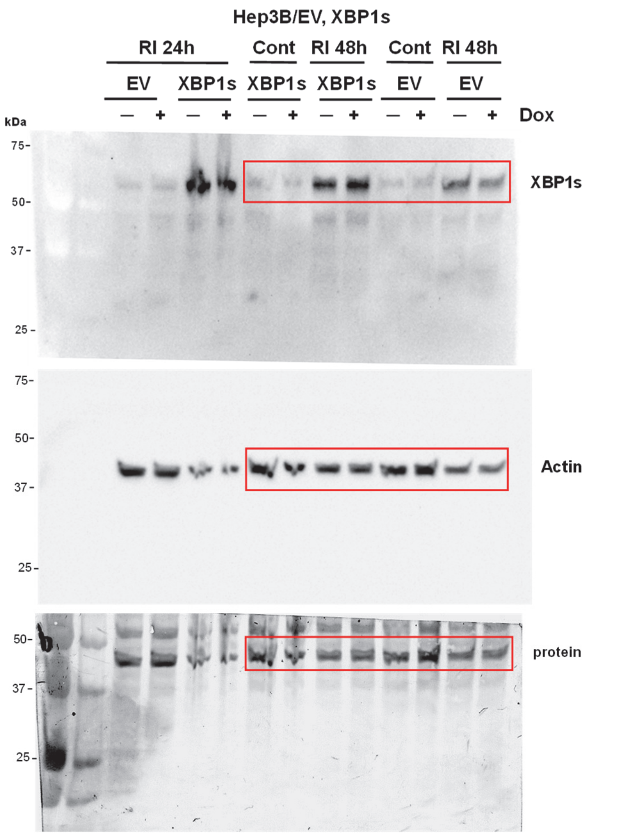

Western blot 8/16/2023

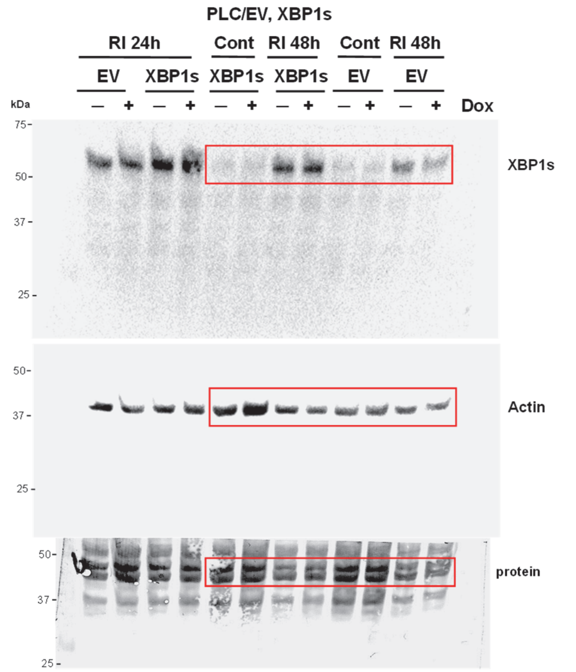

Western blot 8/17/2023

Figure 5D

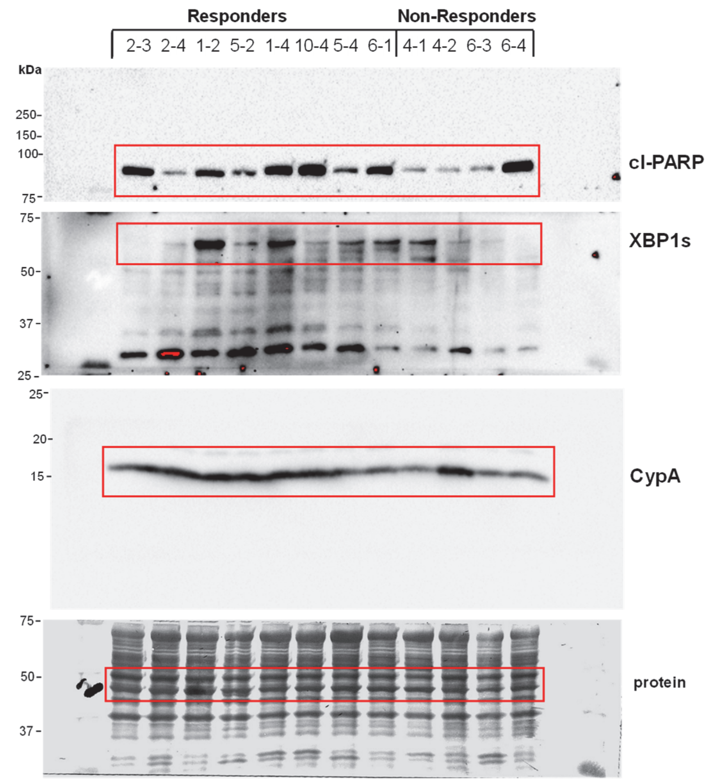

Western blot #1: 4/18/2023

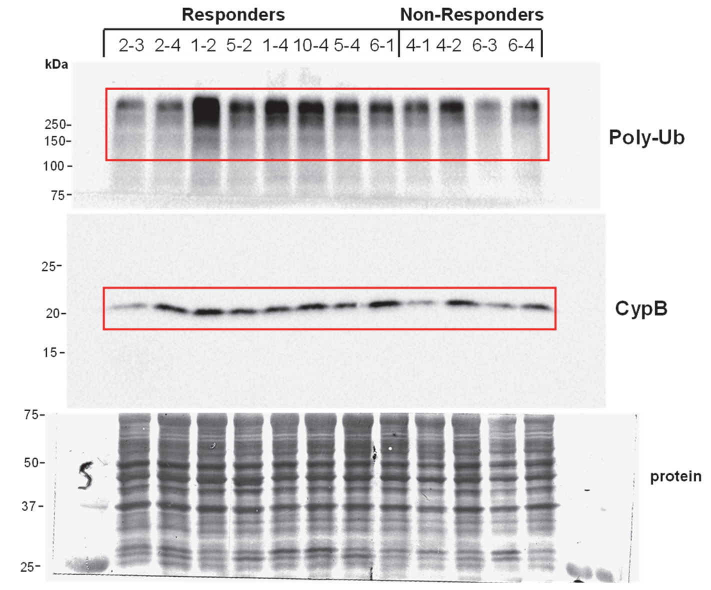

Western blot #2: 4/18/2023

Uncropped Western Blots: Supplementary Figures 1-3, 5-7, 9-11

1. **Blots were cut horizontally to analyze high, medium, or low molecular weight target proteins.**
2. **Regions used in figures are contained within red rectangles.**

Figure S1B

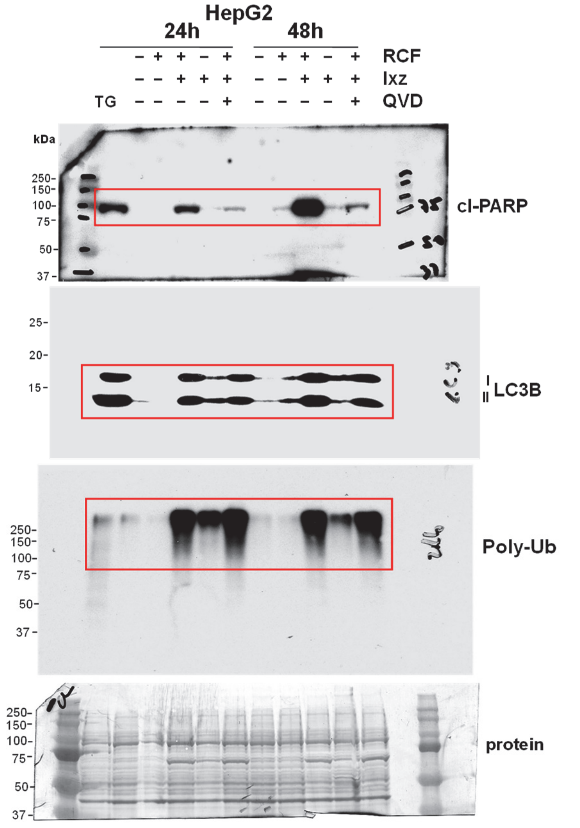

Western blot 11/16/2018

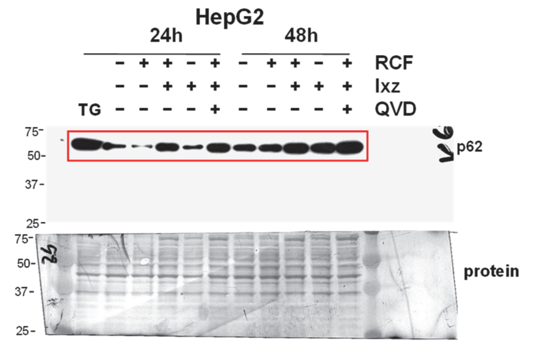

Western blot #2: 11/20/2018

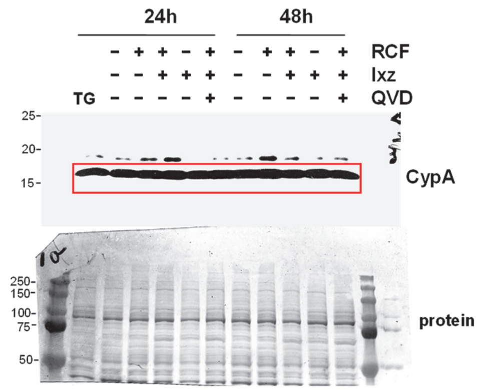

Western blot 11/28/2018

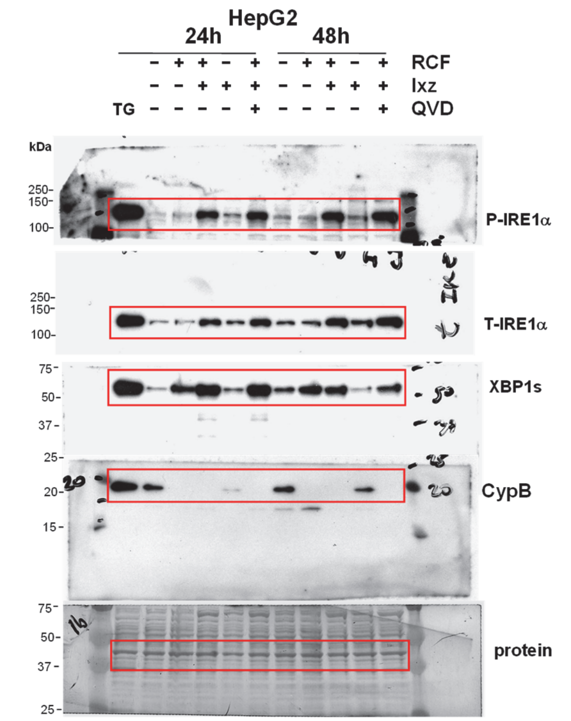

Western blot #1: 11/20/2018

Figure S2A

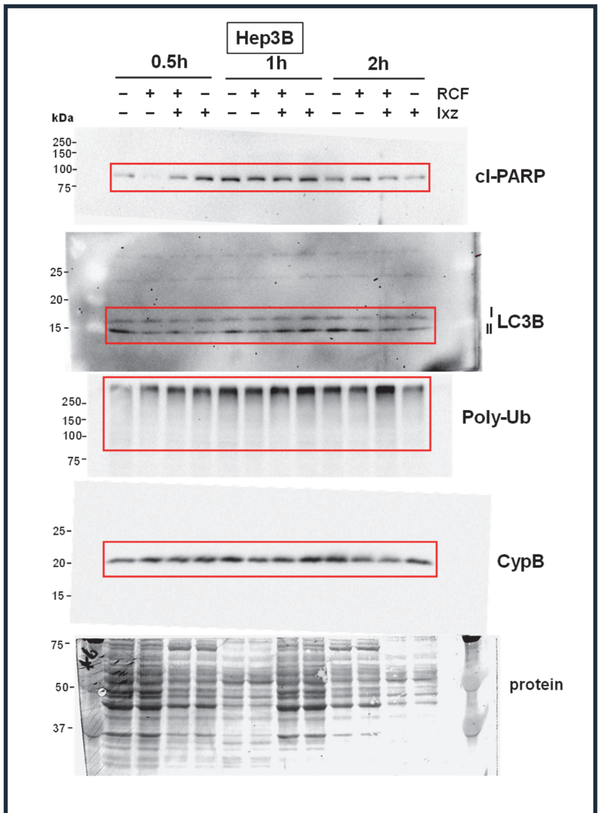

Western blot #1: 8/25/2023

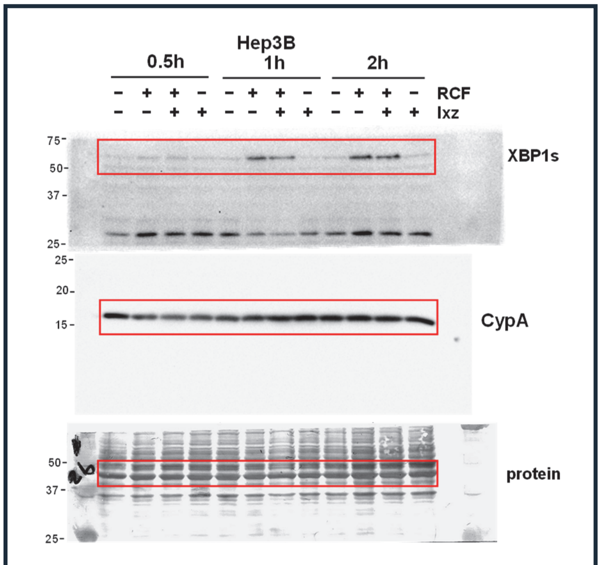

Western blot #1: 9/1/2023

Figure S2B

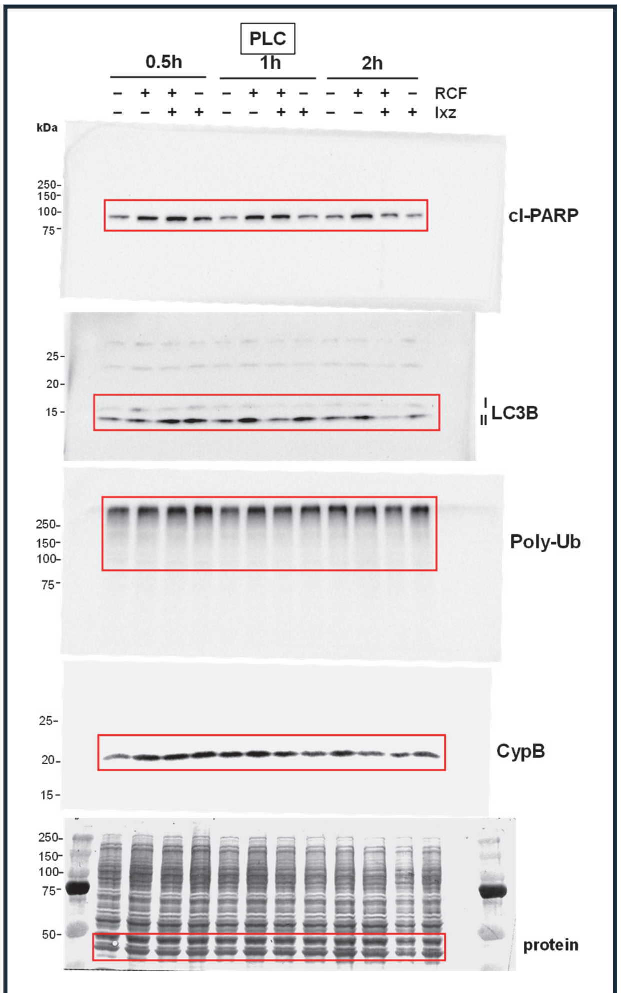

Western blot #2: 8/25/2023

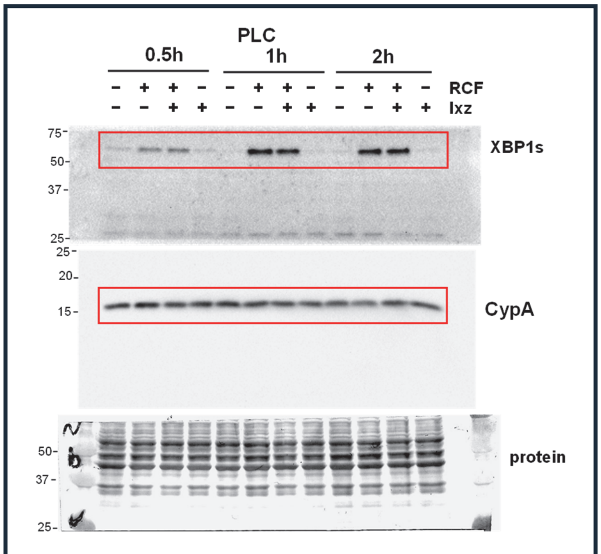

Western blot #2: 9/1/2023

Figure S2C

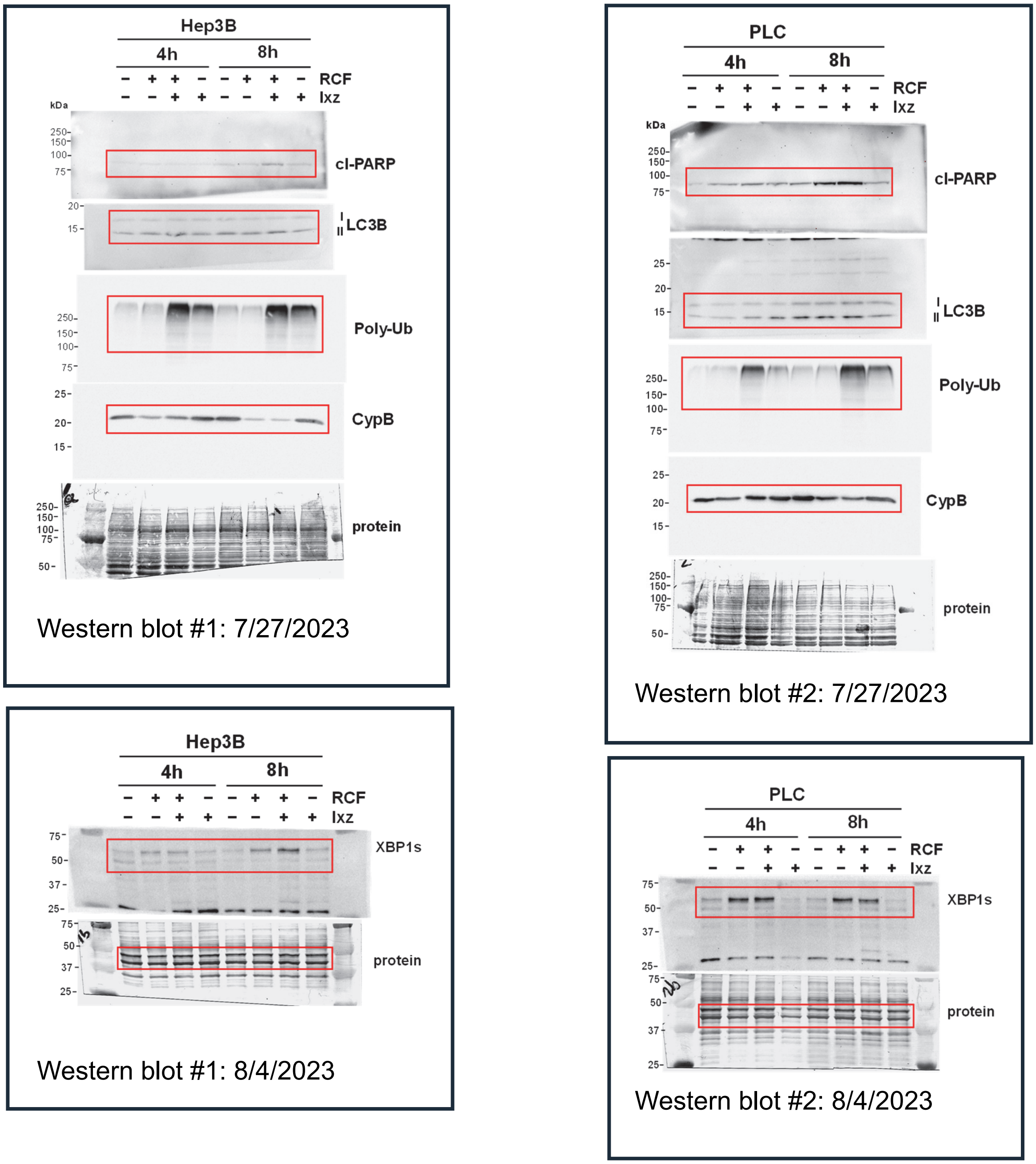

Hep3B
4h
8h
RCF
Ixz
kDa
cl-PARP
LC3B
Poly-Ub
CypB
protein
Western blot #1: 7/27/2023
PLC
Western blot #2: 7/27/2023
XBP1s
Western blot #1: 8/4/2023
Western blot #2: 8/4/2023

Figure S3

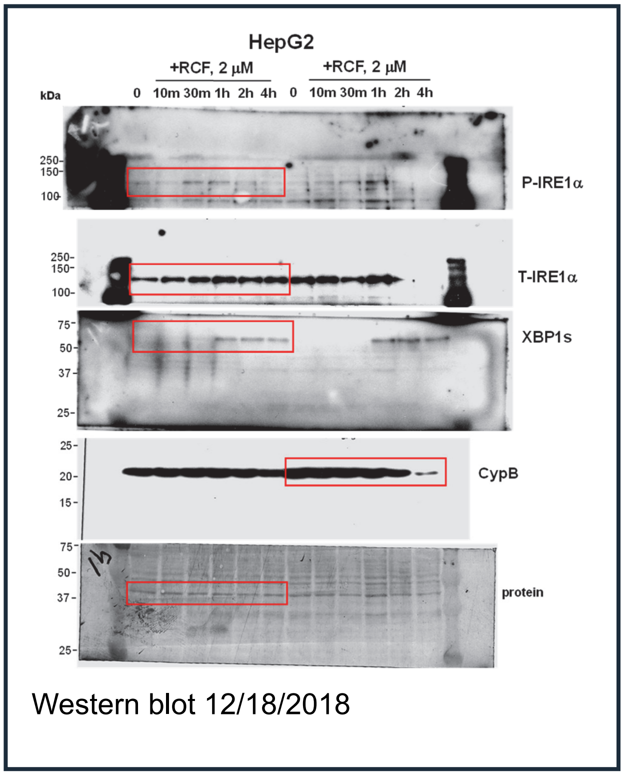
HepG2
+RCF, 2 µM
+RCF, 2 µM
kDa
0 10m 30m 1h 2h 4h 0 10m 30m 1h 2h 4h
P-IRE1α
T-IRE1α
XBP1s
CypB
protein
Western blot 12/18/2018

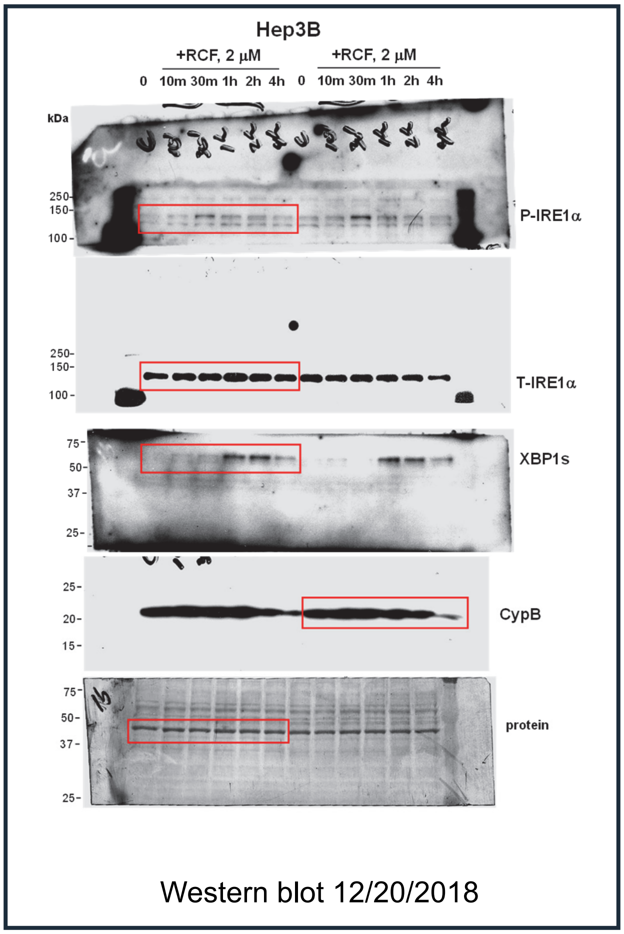
Hep3B
+RCF, 2 µM
+RCF, 2 µM
0 10m 30m 1h 2h 4h 0 10m 30m 1h 2h 4h
kDa
P-IRE1α
T-IRE1α
XBP1s
CypB
protein
Western blot 12/20/2018

Figure S5B

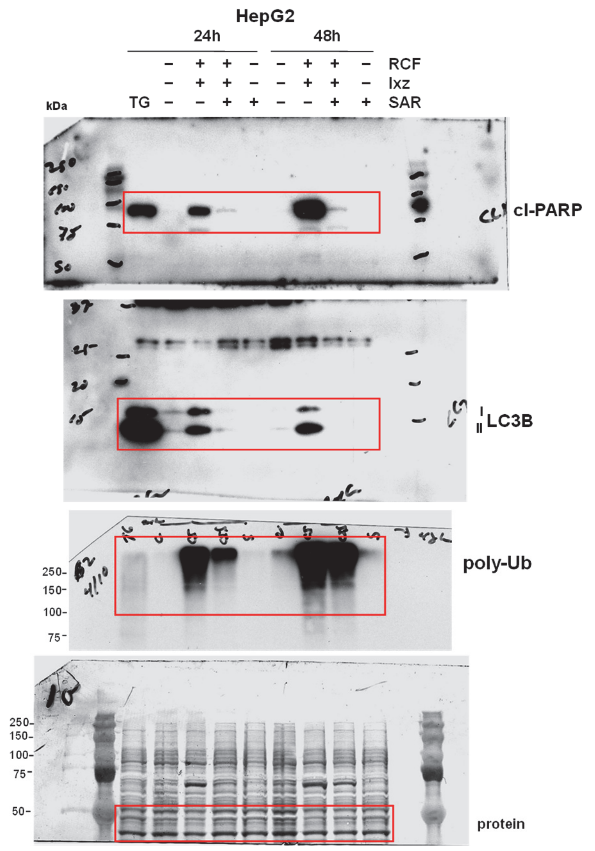

Western blot 8/15/2018

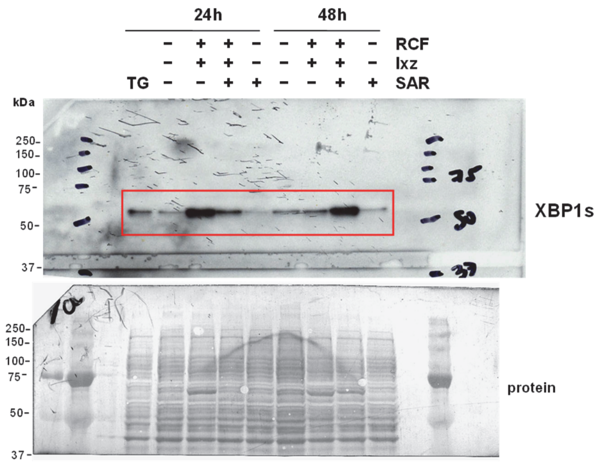

Western blot 11/2/2018

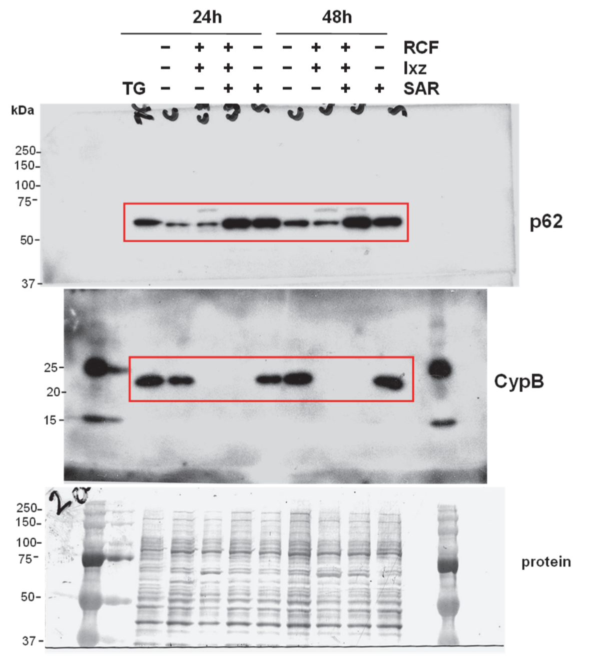

Western blot 8/24/2018

Figure S6B

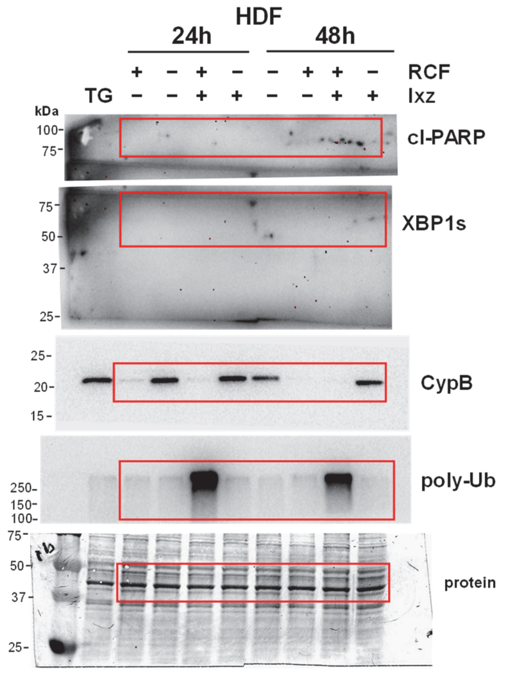

Western blot 6/8/2022

Figure S7

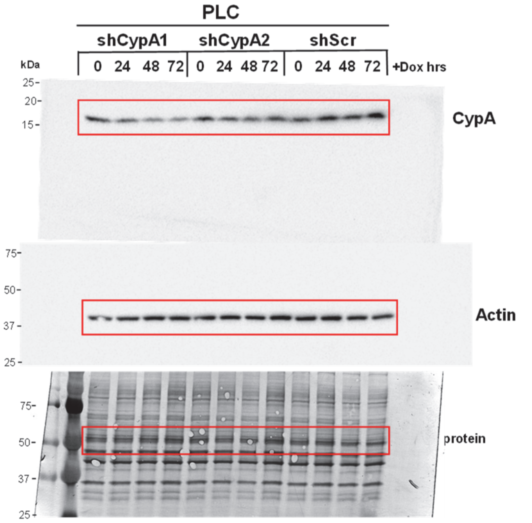

Western blot 5/8/2024

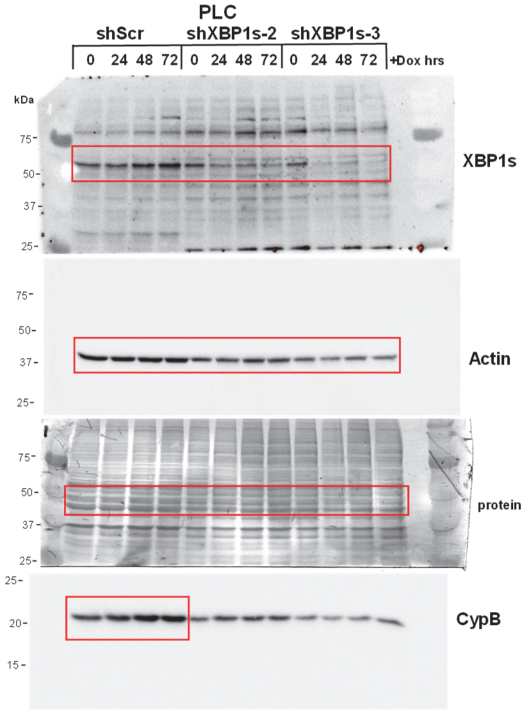

Western blot 5/31/2024

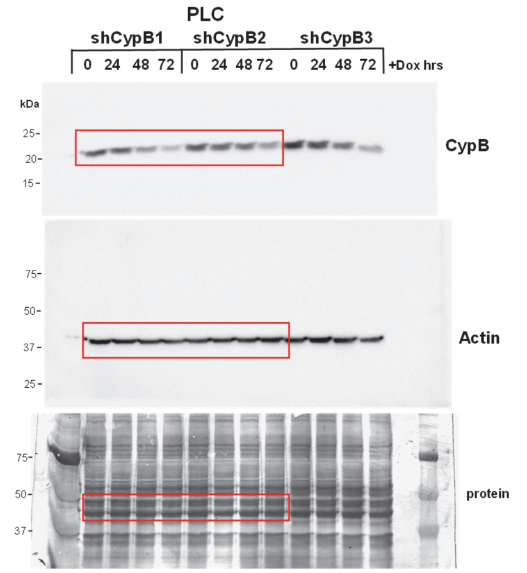

Western blot 5/16/2024

Figure S9

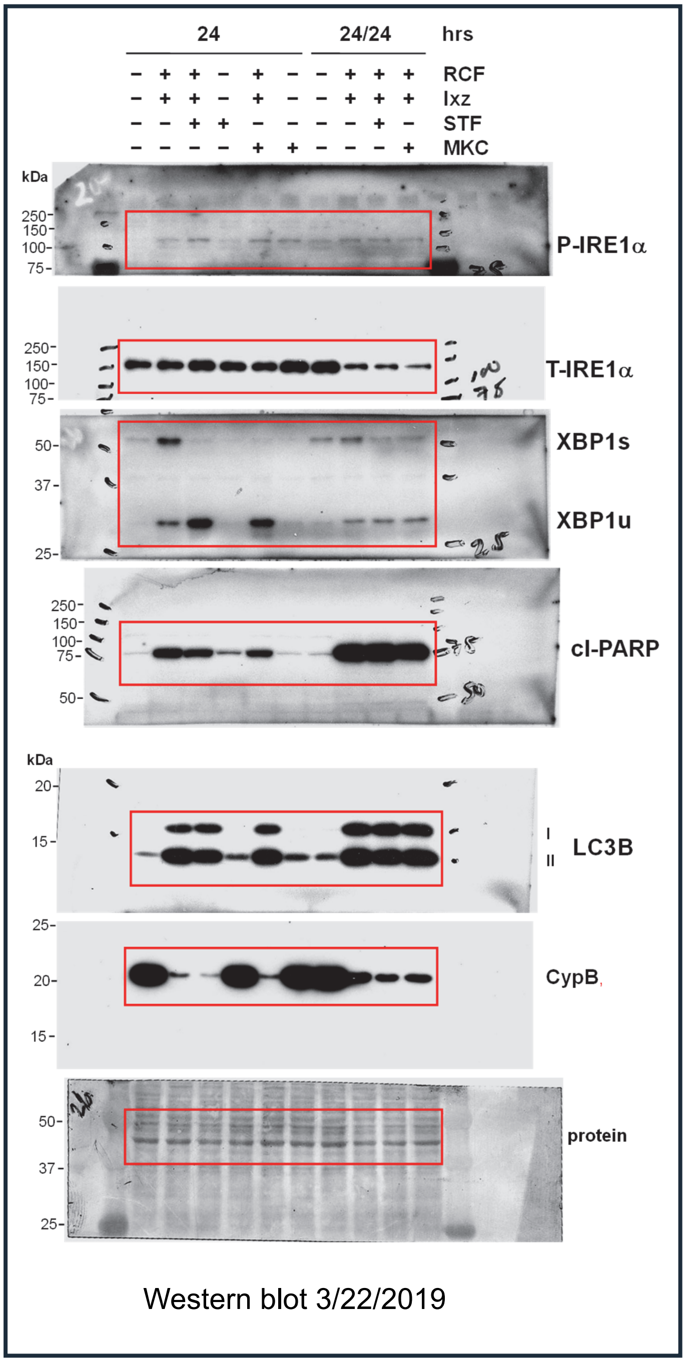

Western blot 3/22/2019

Figure S10

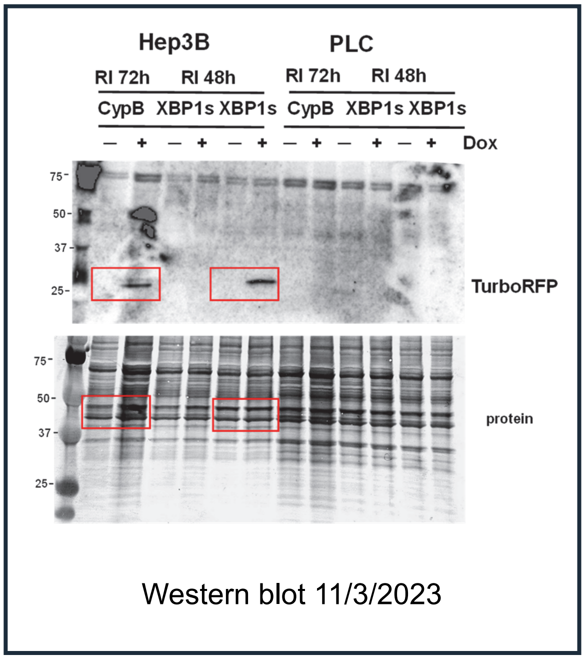

Western blot 11/3/2023

Figure S11

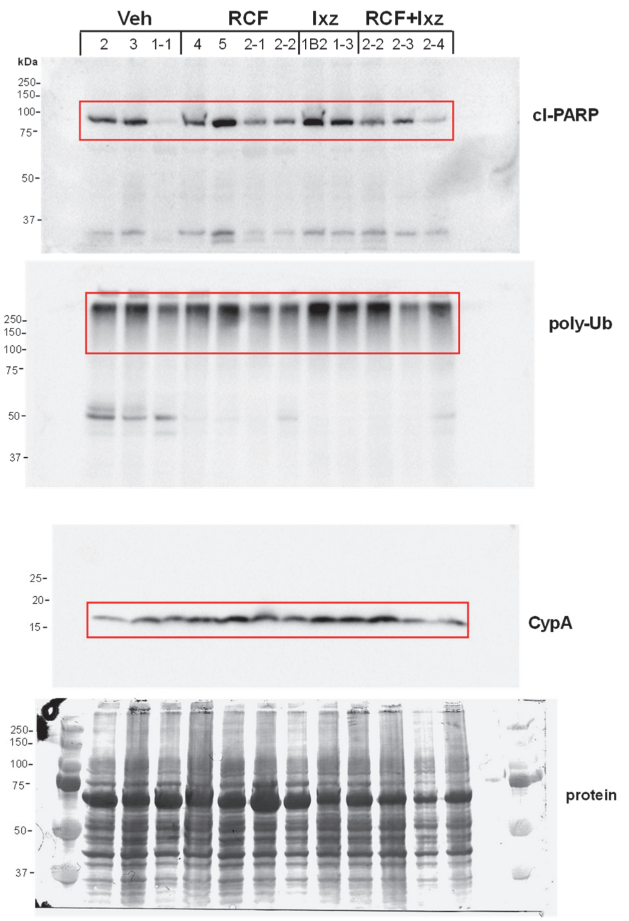

Western blot #1: 11/8/2023

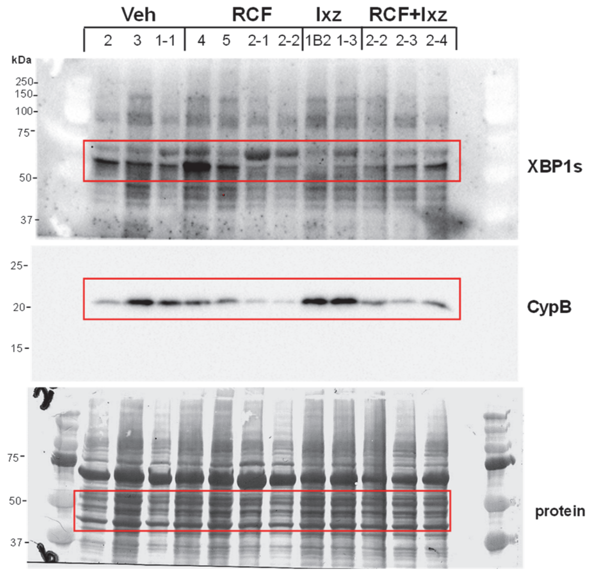

Western blot #2: 11/8/2023
